# CHOOSE TO MOVE: IMPLEMENTATION OF A PHYSICAL ACTIVITY INTERVENTION AT SCALE ACROSS BRITISH COLUMBIA, CANADA

**DOI:** 10.1093/geroni/igz038.447

**Published:** 2019-11-08

**Authors:** Joanie Sims Gould, Heather McKay, Samantha Gray, Adrian Bauman, Lindsay Nettlefold, Christa Hoy

**Affiliations:** 1 University of British Columbia, Vancouver, British Columbia, Canada; 2 University of Sydney, Sydney, NSW, Australia

## Abstract

Despite the many benefits of physical activity (PA), older adults remain among the least active Canadians. Regular PA effectively enhances social connectedness which in turn, is linked to positive health benefits. PA also promotes older adult’s physical mobility which is “the best guarantee of retaining independence and being able to cope” in later years. Although effective PA interventions exist, all but five were conducted at small scale. None were effectively scaled up and sustained over the longer term. To improve population health, effective interventions must be scaled-up. In 2015, BC Ministry of Health released a PA strategy and action plan--older adults were identified as one priority area. In partnership with government and community stakeholders we were entrusted to co-design, implement and evaluate a 6 month, evidence- and choice-based PA intervention (Choose to Move; CTM) across BC, Canada. Implementation and adaptation frameworks and processes we adopted were embedded within socioecological models. We evaluated CTM at scale-up in 26 communities with 458 low active older adults. Our implementation evaluation showed that relationships and infrastructure were key facilitators to delivering CTM at scale. Our impact evaluation showed that PA and social connectedness were enhanced; mental health (loneliness/happiness), grip strength and mobility all improved following participation in CTM. A flexible, adaptable PA model, designed with scalability in mind is key to enhance health indicators in low active older adults. Effectively engaging stakeholders at multiple levels in the implementation process is essential to success.

